# The effect of virtual interactive nurse-led support group intervention on fatigue, shock anxiety, and acceptance of implantable cardioverter defibrillator patients: a randomized trial

**DOI:** 10.1186/s12872-024-03713-5

**Published:** 2024-01-11

**Authors:** Masoume Rambod, Samira Rohaninasab, Nilofar Pasyar, Mohammad Hossein Nikoo

**Affiliations:** 1 Community Based Psychiatric Care Research Center, School of Nursing and Midwifery, Shiraz University of Medical Sciences, Zand St., Nemazee Sq., Shiraz, 7193613119 Iran; 2 Student Research Committee of Shiraz University of Medical Sciences, Shiraz, Iran; 3 Clinical Cardiac Electrophysiology, Cardiovascular Research Center, Cardiology department, Shiraz University of Medical Sciences, Shiraz, Iran; 4 Non-Communicable Disease Research Center, Shiraz University of Medical Sciences, Shiraz, Iran

**Keywords:** Anxiety, Defibrillators, Implantable, Fatigue, Internet based intervention

## Abstract

**Background:**

Implantable cardioverter defibrillators (ICD), as a gold and standard treatment for fatal cardiac arrhythmia, may lead to some physical and psychological problems for the patients. Therefore, performing some interventions to reduce or eliminate these issues is crucial. This study aimed to determine the effect of virtual interactive nurse-led support group intervention on fatigue, shock anxiety, and acceptance of ICD patients.

**Methods:**

This is a clinical trial study on 72 patients with ICD. They were randomly allocated to the intervention (*n* = 36) and control (*n* = 36) groups. A virtual interactive nurse-led support group intervention through WhasApp was performed for one month. Multidimensional fatigue inventory, Florida Shock Anxiety Scale, and Florida Patient Acceptance Scale were used. Data were analyzed to perform the analysis of data through SPSS, using independent and paired-t test, Mann-Whitney U test, Wilcoxon test, and ANCOVA.

**Results:**

Before the intervention, no significant difference was observed between the two groups with regard to fatigue, shock anxiety, and ICD acceptance. However, after the intervention, a significant difference was found between the two groups with regard to fatigue, shock anxiety, and ICD acceptance (*P* < 0.05).

**Conclusion:**

This study showed that virtual interactive nurse-led support group intervention reduced fatigue and shock anxiety and improved the ICD acceptance.

**Practice implications:**

This flexible, accessible, and interactive nurse-led support group intervention is suggested to be used for ICD patients.

**Trial registration:**

This trial was registered and approved by Iranian Registry of Clinical Trials (Trial Id: 60,738, date: (24/02/2022). (https://www.irct.ir/trial/60738).

## Introduction

Cardiovascular diseases are the main cause of death worldwide [1]. Ventricular arrhythmia is one of the most dangerous and sudden deadly type of cardiac arrhythmia [2]. It consists of ventricular tachycardia (VT) and ventricular fibrillation (VF). VT is an arrhythmia of ventricular origin with a heart rate of more than one hundred beats per minute in electrocardiogram (ECG) [3]. VF happens when a ventricular rate is higher than 300 QRS complexes on ECG with irregular electrical activity that does not lead to ventricular contraction [4]. The implantable cardioverter defibrillator (ICD) is the most effective treatment for these types of arrhythmia [2].

Although ICD is the gold and standard treatment for fatal cardiac arrhythmia, it leads to complications [5]; therefore, some of the patients with ICD have lower physical and mental quality of life [6]. Among ICD patients, the dimensions of quality of life and energy/fatigue have been reported in a moderate level [7]. Fatigue was associated with poor sleep [8], depression, anxiety, and stress [7].

These patients also experienced a moderate level of anxiety [9] and shock anxiety [7]. The experience of receiving a shock in patients can be painful; it causes disturbance in their daily life [10] and increases the risk of mortality in ICD patients [11]. In a qualitative study, it was reported that some patients had a positive, yet unrealistic, perspective to ICD; the others had negative views about ICD [12]. On the other hand, in another study, it was indicated that most ICD patients had a positive view and acceptance to ICD [13], and another one revealed that ICD acceptance was in a moderate level [7].

As mentioned above, patients with ICD face challenges such as fatigue and shock anxiety, and contradictory findings regarding the acceptance of the device have been reported. Moreover, more than half of ICD patients had no knowledge regarding this device, and they were moderately concerned in this regard [14]. Therefore, it is necessary to pay attention to these challenges.

In this study, we considered the use of virtual interactive nurse-led support group intervention. In fact, providing a support group intervention and helping to increase the ICD patients’ understanding of the disease and device by professional healthcare workers, especially nurses, are crucial [15] because the literature review showed social support was associated with compliance and quality of life in cardiac patients [16]. However, few studies have focused on virtual interactive nurse-led support group intervention; in fact, they have used some parts of this intervention. For example, it was reported that web-based educational support intervention was effective in coronary heart disease patients [17], and an Internet training program was effective in psychological symptoms [18]. Patient-assisted computerized education for ICD patients reduced their trait anxiety and improved the acceptance of the device [19]. These studies used online or computerized intervention [17, 19]. Other studies focused on psychological-educational intervention and indicated that it improved the physical dimension of the quality of life [20] and device acceptance in ICD patients and reduced shock anxiety in these patients [19]. Two other studies have focused on nurse-led based intervention; although this intervention was not conducted on ICD patients, it was reported it reduced fatigue in patients with chronic disease [21, 22]. Moreover, a limited number of studies have focused on group interventions [23], and virtual health educators support group intervention [24] in other chronic conditions.

In fact, the limitation of the above studies is that if the intervention is done only online, the person must be present during the implementation of the program and daily busyness can affect the person’s online presence. The problem with using computerized intervention is that not all people have access to this device. In addition, using CD-ROMs is not common these days. However, it can be said that a large percentage of people these days have smart phones. They use social networks to establish social relationships, share their interest and hobbies, stay in touch with the world, and are able to communicate with each other anywhere. Therefore, the use of virtual intervention using social networks can be a strong point. Furthermore, based on the researchers’ experience, interactive nurse-led support group actively encourages the ICD patients to participate in the program daily, sets up a support group, and get involved in their learning process.

As mentioned, the current studies implemented a part of virtual interactive nurse-led support group intervention, and there was limited information about the effect of these interventions on fatigue, shock anxiety, and acceptance of patients with ICD. Therefore, this study aimed to determine the effect of virtual interactive nurse-led support group intervention on fatigue, shock anxiety, and acceptance of ICD patients.

## Methods

### Design

This randomized controlled clinical trial study was conducted on a parallel group (an intervention and a control group). This study was conducted from April to May 2022 and was registered and approved by Iranian Registry of Clinical Trials (Trial Id: 60,738, date: (24/02/2022).

### Setting

The setting of this study was a pacemaker and ICD center of Shahid Faghihi hospital affiliated to Shiraz University of Medical Sciences (SUMS), Shiraz, Iran.

### Participants

The inclusion criteria were passing at least 6 months of the implantation of ICD; being 18 years old or more; being able to speak Persian; being oriented to time, person, and place; and having access to the Internet and social networks (WhatsApp). The patients who were a known case of mental health disorders such as depression, anxiety, psychosis, etc. had major crises such as the death of the loved ones and had got divorce during the past 6 months, were unwilling to continue cooperation, and had not participated in the intervention program completely for more than 3 consecutive days and had not made up for it until one week later were excluded.

**Fig. 1:**
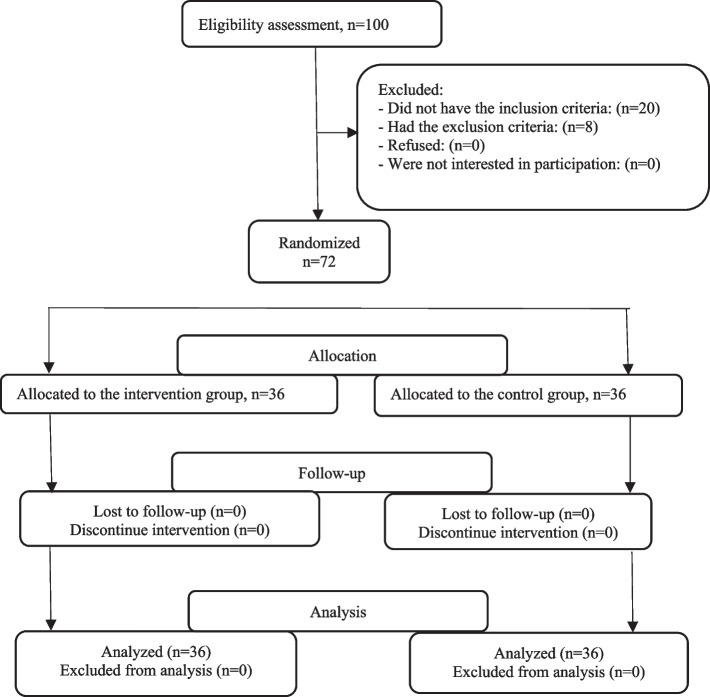
Flow chart of the ICD patients who participated in this study

As Fig. 1 shows, one hundred subjects were assessed for eligibility to participate in this study; 15 patients did not meet the inclusion criteria, and 13 participants were excluded. Thus, 72 patients were allocated to the intervention and control groups. As Fig. 1 shows, all subjects completed the study.

### Sample size

The sample size was estimated based on a pilot study, dropout rate of 20%, α of 0.05, β of 0.90, shock anxiety (µ1–µ2 = 9.40), δ of 9.60, patients’ acceptance (µ1–µ2 = 17.90), δ of 19.20, and fatigue (µ1–µ2 = 10.80), δ of 10.10. The sample size was calculated as 36, 35, and 36 in each group based on shock anxiety, patients’ acceptance, and fatigue results, respectively. Therefore, based on higher sample size, it was estimated 72 subjects (36 in each group).

### Randomization

First, 72 patients who had records in the pacemaker and ICD center were selected using a random number table. Then, random allocation software was used to create 18 blocks with a block size of four. Then, based on the list generated by this software (BABA, ABBA, BAAB, BABA, AABB, etc.), the patients were allocated to the intervention (B) and control (A) groups. Then, each block was placed in a sealed sequentially numbered envelope by someone outside the research team. After choosing the person using a random number table, we opened the envelope sequentially based on its content (A or B); the patient was allocated to the intervention or control groups.

### Blinding

The individuals who collected and analyzed the data were blind to the study groups. Moreover, the healthcare providers (physicians, nurses in the pacemaker, and ICD center) were blind to the study groups.

### Measurements

The outcomes were measured before and four weeks after the intervention. The study data were collected using a demographic information form containing information on age, gender, educational level, and marital status. In addition, the patients were asked for information about the ICD including the duration of having an ICD, whether they received shocks (Yes/No), and total number of shocks received. Moreover, ejection fraction (EF), number of recreational and travel events after implantation of an ICD, and hours of walking per week since implantation of an ICD were assessed.

The multidimensional fatigue inventory is a self-report instrument used to measure fatigue. It has 20 items and 5 domains: general fatigue (4 items), physical fatigue (4 items), mental fatigue (4 items), reduced activity (4 items), and reduced motivation (4 items). The responses are scored based on a 5-point Likert scale ranging from “yes, absolutely true” to “no, absolutely false”. A score of 1 to 5 is calculated for each item, and in some items, reverse scoring is done; therefore, the total score of each field is 4˗20, and the total score of fatigue as determined by the sum of the scores of the fields is 20–100. A higher score indicates more tiredness of the person. Smets et al. reported the reliability of this inventory as 0.84, and its validity was between 0.92 and 0.78 [25]. The reliability of the Persian version of this inventory was approved using Cronbach’s alpha (0.75) [26]. In this study, the internal consistency of the inventory was reported 0.78, using Cronbach’s alpha coefficient.

Florida Shock Anxiety Scale (FSAS): This scale was designed by Kuhl et al. for patients with ICDs in 2006 to assess ICD-specific anxiety and the cognitive, behavioral, and emotional effects of shock. FSAS contained 10 items scored based on a 5-point Likert scale ranging from 1 (never) to 5 (always). Thus, the total score of the scale could range from 10 to 50, with higher scores indicating higher shock anxiety levels. In the original study, the validity and reliability of FSAS were confirmed, and its Cronbach’s alpha coefficient was 0.91 [27]. In this study, the internal consistency of FSAS was approved, using Cronbach’s alpha coefficient (0.88).

Florida Patient Acceptance Scale (FPAS): This scale was first developed by Burns et al. in 2005 to evaluate the acceptance of patients with pacemakers and ICDs. Its short form included 12 items and 3 subscales, namely device-related distress, positive appraisal, and return to functioning, each containing four items. The items were scored based on a Likert scale ranging from one (totally disagree) to five (totally agree), with higher scores representing better acceptance of the device [28]. The total scores of acceptance and its subscales were linearly converted to a score between 0 and 100. The validity of the short form was approved by Versteeg et al., and its reliability was confirmed by Cronbach’s alpha coefficient of 0.76 [29]. In this study, the internal consistency of FSAS was approved using Cronbach’s alpha coefficient (0.86).

### Intervention

The intervention was done through 26 MP4 video files in the form of micro-teaching using WhatsApp. It led by an MS nurse for four weeks according to the schedule. The duration of each file was 3˗10 min, which could be easily uploaded and downloaded on WhatsApp. Their size was also small, so that it could be opened in mobile phones with low capacity. Table 1 shows the contents of the videos presented in this study.

**Table 1:** The contents of videos presented in this study

The importance of social support, group relationships, and communications; and family, friends, peers, healthcare providers, and nurses’ social support in patients with ICD
The way to change their relationship and roles while they are living with ICD
Anatomy and physiology of the heart, arrhythmia, ICD device, and the way it functions; and preparation before, during, and after ICD implant procedure and checkup
ICD precautions and care during driving, sports, work and daily life; and the method of taking their pulse
The feelings, emotions, and body image and self-image concerns in living with ICD, the way to cope with and manage stress, fear, worries and psychological issues, as well as the way to keep calm and relaxed and think positively.
Spiritual strategies such as prayer, gratitude to God, and communication with God to maintain peace and relaxation in ICD patients

In addition to the MP4 videos provided, the WhatsApp channel was opened every day for one hour, so that the subjects could chat and communicate together under supervision of the third author of this study. These hours were announced the day before, and the subjects could ask their questions and interact with each other. During this time, the participants expressed their questions and shared their experiences regarding the occurrence of shock, shock management, their feelings, and strategies to cope with these sensations and stress. They shared their information regarding the information about the device, such as the battery life of ICD, and the physician and ICD clinic follow-up. Other topics discussed by the participants were ICD precautions, job restrictions, physical activity, and exercise. Moreover, the subjects communicated with the researcher (the third author who was an MS nurse) of this study in personal WhatsApp in the form of text and voice messages, and audio call in specific times a day. The control group received routine care by pacemaker and ICD center.

### Ethical considerations

This study was approved by the Ethics Committee of Shiraz University of Medical Sciences (Code: IR.SUMS.NUMIMG.REC.1400.068). The study objective was explained to the patients who were asked to sign written informed consent for taking part in the research. They were reassured of the confidentiality of their information and the voluntary nature of the study.

### Data analysis

The data were analyzed using SPSS statistical software, version 22. Independent t-test and chi-square test were used to assess the differences between the two groups regarding demographic characteristics. Additionally, independent t-test and Mann-Whitney U test were used to compare the study groups’ pre-and post-intervention. Paired t-test was also used. Moreover, ANCOVA was used to control the effect of the confounding variables on the post-test. In this study, the duration of implantation of ICD, number of shocks received, and EF were considered as the confounders. *P* < 0.05 was considered as statistically significant.

## Results

### Demographic and clinical characteristics of the ICD patients

The mean age of the ICD patients was 45.42 (SD = 10.98) and 49.61 (SD = 9.02) years in the intervention and control groups, respectively. As Table 1 shows, more than half of the subjects in both groups were male and married and had primary and high school education.

As shown in Table 2, the mean duration of having an ICD was 22.06 (SD = 7.96) and 19.06 (SD = 6.06) months in the intervention and control groups, respectively. Half of the subjects in the intervention group and 58.3% of the participants in the control group had received at least one shock. The number of shocks received since ICD implantation was 2.75 (SD = 4.81) and 1.94 (SD = 3.81) in the intervention and control groups, respectively. 61.1% of the participants of both groups had experienced shock during anger and stress. Most of the ICD patients in both intervention and control groups had an EF lower than 40%.

**Table 2:** Demographic and clinical characteristics of the ICD participants in the intervention and control groups

Variables	Groups		Test, *p*-value
	Intervention	Control	
Gender, n (%)			
Male	22 (61.1)	26 (72.2)	χ^2^ = 1.00
Female	14 (38.9)	10 (27.8)	*P* = 0.31
Education level, n (%)			
Primary school	10 (27.8)	15 (41.7)	χ^2^ = 3.38
Secondary school	9 (25.0)	10 (27.8)	*P* = 0.33
High school and Diploma	9 (25.0)	8 (22.2)	
Academic	8 (22.2)	3 (8.3)	
Marital status, n (%)			
Single	4 (11.1)	4 (11.1)	χ^2^ = 0.00
Married	31 (86.1)	31 (86.1)	*P* = 1.00
Widow	0(0.0)	1 (2.8)	
Divorced	1 (2.8)	0 (0.0)	
Ejection Fraction, n (%)			
< 20%	7 (31.8)	11 (45.8)	χ^2^ = 4.70
20–30%	6 (27.3)	2 (8.3)	*P* = 0.31
30–40%	1 (4.5)	4 (16.7)	
40–50%	2 (9.1)	2 (9.1)	
> 50%	6 (27.3)	5 (20.2)	
Receiving a shock from the implantation of an ICD, n (%)			
Yes	18 (50.0)	21 (58.3)	χ^2^ = 0.50
No	18 (50.0)	15 (41.7)	*P* = 0.47
Time of ICD shock, n (%)			
In sleep	2 (11.1)	3 (16.7)	χ^2^ = 2.98
When I am sitting and doing nothing	3 (16.7)	1 (4.8)	*P* = 0.39
During physical activity and housework	2 (11.1)	6 (28.6)	
In times of stress and anger	11 (61.1)	11 (61.1)	
Duration of implantation of ICD^a^, Mean (SD)	22.06 (7.96)	19.06 (6.06)	Z^‡^= − 1.72 *P* = 0.08
The number of received shocks, Mean (SD)	2.75 (4.81)	1.94 (3.81)	Z^‡^= − 0.54 *P* = 0.54
Number of recreational and travel events after implantation of an ICD, Mean (SD)	5.61 (9.09)	7.56 (12.16)	Z^‡^= − 0.25 *P* = 0.79
Hours of walking per week since implantation of an ICD, Mean (SD)	3.25 (2.83)	2.67 (2.05)	Z^‡^= − 0.53 *P* = 0.59

^a^Months

^‡^Mann-Whitney U test

As displayed in Table 2, the number of recreational and travel events after implantation of an ICD was 5.61 (SD = 9.09) and 7.56 (SD = 12.16) in the intervention and control groups. Moreover, hours of walking per week since implantation of an ICD was 3.25 (SD = 2.83) and 2.67 (SD = 2.05) in the intervention and control groups, respectively.

### The effect of the intervention on fatigue in patients with ICD

**Fig. 2:**
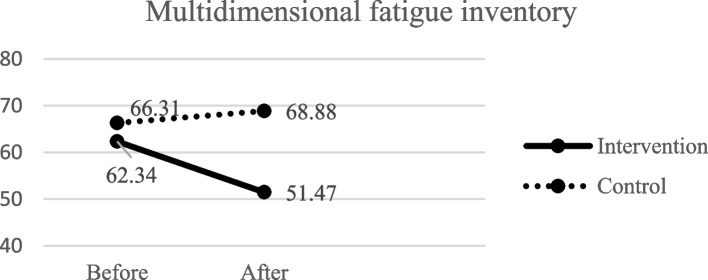
The two groups’ multidimensional fatigue inventory scores before and after the intervention

As shown in Table 3; Fig. 2, before the intervention, the mean score of fatigue was 62.34 (SD = 13.96) and 66.31 (SD = 14.47) in the intervention and control groups, respectively. Also, before the intervention, independent t-test showed that no significant difference was observed between the two groups with regard to fatigue and five domains (*P* > 0.05). However, after the intervention, the mean score of fatigue was 51.47 (SD = 6.44) in the intervention group and 69.06 (SD = 13.38) in the control group. ANCOVA showed that a significant difference was found between the two groups regarding the mean score of fatigue and its domains (*P* < 0.05).

**Table 3:** Comparison of the mean scores of fatigue and its dimensions and shock anxiety in the intervention and control groups before and after the intervention

Variables	Groups		Test, *p*-value
	Intervention Mean (SD)	Control Mean (SD)	
Multidimensional fatigue inventory			
Before	62.34 (13.34)	66.31 (14.47)	t^†^ = -1.11, 0.29
After	51.47 (6.44)	69.06 (13.38)	F^‡^ = 24.29, < 0.001
Test^I^, *p*-value	3.75, < 0.001	-2.78, 0.005	
**Fatigue dimensions**			
General fatigue			
Before	13.58 (2.82)	14.11 (3.62)	
After	9.91 (1.91)	14.36 (2.92)	t^†^ = − 0.68, 0.49
Test^I^, *p*-value	4.37, < 0.001	− 0.51, 0.60	F^‡^ = 36.71, < 0.001
Physical fatigue			
Before	12.57 (2.98)	13.52 (2.96)	t^†^ = − 1.33, 0.18
After	11.97 (2.15)	15.02 (2.89)	F^‡^ = 16.60, < 0.001
Test^I^, *p*-value	-1.03, 0.30	-3.21, 0.001	
Mental fatigue			
Before	11.91 (3.83)	12.05 (3.57)	Z^II^ = − 0.01, 0.99
After	10.88 (1.76)	14.00 (4.13)	F^‡^ = 6.38, 0.01
Test^I^, *p*-value	− 1.94, < 0.05	-2.04, 0.04	
Reduced activity			
Before	12.60 (4.20)	13.61 (4.51)	Z^II^ = -0.80, 0.41
After	14.25 (3.36)	16.25 (3,37)	F^‡^ = 6.69, 0.01
Test^I^, *p*-value	1.39, 0.17	-1.17, 0.24	
Reduced motivation			
Before	11.11 (3.30)	12.30 (3.93)	Z^II^ = − 1.35, 0.17
After	9.61 (2.12)	12.22 (3.89)	F^‡^ = 5.67, 0.02
Test^I^, *p*-value	2.25, 0.02	− 1.85, 0.06	
Shock anxiety			
Before	30.25 (10.67)	30.42 (9.66)	F^‡^ = 0.01, 0.91
After	24.40 (8.65)	33.19 (8.57)	F^‡^ = 14.23, < 0.001
Test ^III^, *p*-value	4.62, < 0.001	-9.55, < 0.001	

^I^Wilcoxon test

^†^Independent t-test

^‡^ANCOVA, duration of implantation of ICD, number of received shocks and Ejection fraction as the covariates

^II^Mann-Whitney U test

^III^Pair t-test

### The effect of the interventions on shock anxiety in patients with ICD

**Fig. 3:**
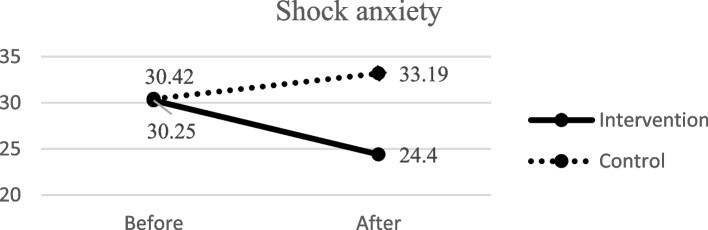
The two groups’ mean score of shock anxiety before and after the intervention

Moreover, there was no significant difference between the two groups regarding shock anxiety before the intervention using ANCOVA (F = 0.01, *P* = 0.91). However, ANCOVA indicated that a significant difference was observed between the two groups in shock anxiety after the intervention (F = 14.23, *P* < 0.001) (Table 3; Fig. 3).

### The effect of the intervention on acceptance of patients with ICD

**Fig. 4:**
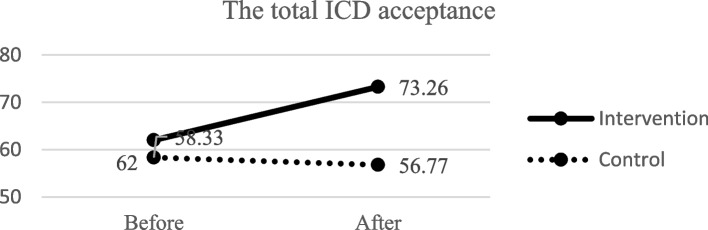
The two groups’ total ICD acceptance score before and after the intervention

As Table 4; Fig. 4 show, before the intervention, there was no significant difference between the two groups regarding the acceptance of ICD and its three subscales using Mann-Whitney U test (*P* > 0.05). On the other hand, after the intervention, ANCOVA showed that a significant difference was observed between the two groups with regard to acceptance of ICD and its subscales, such as positive appraisal and return to normal functioning (*P* < 0.05).

**Table 4:** Comparison of the mean scores of ICD acceptance and its subscales in the intervention and control groups before and after the intervention

Variables	Groups		Test, *p*-value
	Intervention Mean (SD)	Control Mean (SD)	
Total ICD acceptance			
Before	62.00 (19.99)	58.33 (23.18)	Z^†^ = 0.68, *P* = 0.49
After	73.26 (18.47)	56.77 (22.52)	F^‡^ = 6.59, *P* = 0.01
Test^I^, *p*-value	-3.41, 0.001	-1.94, 0.05	
Device-related distress			
Before	45.65 (33.16)	50.17 (32.37)	Z^†^ = 0.62, *P* = 0.53
After	34.54 (30.91)	48.09 (31.26)	F^‡^ = 2.01, *P* = 0.16
Test^I^, *p*-value	-2.56, 0.01	-1.40, 0.15	
Positive appraisal			
Before	78.67 (19.71)	78.29 (20.94)	Z^†^ = -0.13, *P* = 0.89
After	88.36 (16.23)	74.13 (22.76)	F^‡^ = 7.15, *P* = 0.01
Test^I^, *p*-value	-3.13, 0.002	-3.80, 0.002	
Return to functioning			
Before	51.38 (26.22)	46.87 (29.02)	Z^†^ = -0.76, *P* = 0.44
After	65.97 (20.94)	44.27 (26.27)	F^‡^ = 6.70, *P* = 0.01
Test^I^, *p*-value	-3.74, < 0.001	-2.96, 0.003	

^I^Wilcoxon test

^†^Mann-Whitney U test

^‡^ANCOVA, duration of implantation of ICD, number of received shocks and Ejection fraction as the covariates

## Discussion

This randomized clinical trial study indicated that virtual interactive nurse-led support group intervention was effective to reduce fatigue and shock anxiety, and improve acceptance of Iranian implantable cardioverter defibrillator patients.

This study showed that virtual interactive nurse-led support group intervention decreased fatigue in ICD patients. Since no similar study has been conducted, a comparison was made with similar interventions and other chronic diseases. In a systematic review and meta-analysis, it was reported that nurse-led care was an effective intervention in reducing fatigue in patients with chronic disease such as rheumatoid arthritis [21]. In another study, it was indicated that a nurse-led intervention reduced ovarian cancer related fatigue [22]. Group interventions also reduced emotional distress and fatigue in cancer patients [23]. In fact, virtual health educators support group interventions which plays an important role in maintaining healthy behaviors in patients with heart disease [24]. Higher social support is associated with lower physical and mental fatigue in coronary artery disease patients [30]. In our study, the subjects were familiar with the importance of social support; the way to change their physical, psychological, and social relationship; and the change in their roles while living with ICD, the way to cope and manage stress, fear, worries and psychological issues; as well as the way to keep calm and relaxed and think positively. Moreover, they became familiar with spiritual strategies to maintain peace and relaxation. Therefore, this virtual interactive nurse-led support group intervention reduced the ICD patients’ fatigue.

The study showed that virtual interactive nurse-led support group intervention clinically decreased shock anxiety and improved the acceptance in ICD patients. Similarly, it was reported that patient-assisted computerized education for ICD patients reduced trait anxiety and improved the acceptance of the device [19]. In fact, like our study, using psycho-educational interventions which focus on coping, mood, relationships, and device functioning contents [19] helps the ICD patients to experience less shock anxiety and improve device acceptance. It was reported that the Internet training program was effective in psychological symptoms such as depressive symptoms [18]. In fact, patients support group interventions in which people with similar experiences and concerns provide emotional support are taught on coping strategies and inform the patients regarding medical conditions [31]. Moreover, participation in this intervention helps them to get familiar with emotional, physical, financial, and social challenges of a chronic disease and the methods to cope with its treatment [32]. It helps the chronic disease patients to feel empowered [32], improve psychological well-being [33], cope with their similar situation [32], and feel the reduction in their depressive symptoms [34]. Therefore, it seems that these possibly positive changes caused by virtual interactive nurse-led support group intervention have been able to reduce ICD patients’ shock anxiety and promote their acceptance.

The baseline mean scores of shock anxiety in the intervention and control groups were 30.25 (SD = 10.67) and 30.42 (SD = 9.66), respectively, which is high; after the virtual interactive nurse-led support group intervention, although this mean score decreased in the intervention group, its mean score was still high. Therefore, it is suggested that other interventions that can lead to a further reduction in shock anxiety should be carried out in future.

This study was conducted in a pacemaker and ICD center of Shahid Faghihi hospital affiliated to SUMS, Shiraz, Iran; to increase the generalizability, we suggest that further studies should be conducted in other parts of the world.

## Conclusion

This study showed virtual interactive nurse-led support group intervention reduced fatigue and shock anxiety and improved acceptance of ICD patients. Therefore, using virtual interactive nurse-led support group intervention is suggested in ICD patients because of its usefulness in effective time management, access to the intervention from anywhere at any time, flexibility, and cost-effectiveness.

### Practice implications

The implication of this study for practice is that virtual interactive nurse-led support group intervention, through participation of the ICD patients in the virtual group interventions, might help them to feel less lonely and isolated, talk openly and honestly about their chronic conditions, and improve skills to cope with ICD challenges. Moreover, since the virtual intervention is acceptable at any time, is easy and attractive to participate in, and has diverse perspectives, it helps the ICD patients to reduce fatigue and shock anxiety and increase ICD acceptance. It is suggested that the advantages of this virtual interactive nurse-led support group intervention should be clarified to healthcare providers, especially nurses and ICD patients.

One of the strengths of this study was that the intervention was conducted after COVID-19 pandemic; at that time, heart disease patients such as ICD patients were less inclined to attend medical centers, and virtual communication methods had become common among people.

## Data Availability

The data of this study are available from the first author on reasonable request.
